# The XFP (17-BM) beamline for X-ray footprinting at NSLS-II

**DOI:** 10.1107/S1600577519003576

**Published:** 2019-06-04

**Authors:** Awuri Asuru, Erik R. Farquhar, Michael Sullivan, Donald Abel, John Toomey, Mark R. Chance, Jen Bohon

**Affiliations:** a Center for Synchrotron Bioscience, Department of Nutrition, Case Western Reserve University, 10900 Euclid Avenue, Cleveland, OH 44106, USA; b Systems Biology Graduate Program, Medical Scientist Training Program, Case Western Reserve University, 10900 Euclid Avenue, Cleveland, OH 44106, USA

**Keywords:** footprinting, structural dynamics, beamline, solution state

## Abstract

Hydroxyl-radical mediated synchrotron X-ray footprinting (XF) is a powerful solution-state technique in structural biology for the study of macromolecular structure and dynamics of proteins and nucleic acids, with several synchrotron resources available to serve the XF community worldwide. The XFP (Biological X-ray Footprinting) beamline at the NSLS-II was constructed on a three-pole wiggler source at 17-BM to serve as the premier beamline for performing this technique, providing an unparalleled combination of high flux density broadband beam, flexibility in beam morphology, and sample handling capabilities specifically designed for XF experiments. The details of beamline design, beam measurements, and science commissioning results for a standard protein using the two distinct XFP endstations are presented here. XFP took first light in 2016 and is now available for general user operations through peer-reviewed proposals. Currently, beam sizes from 450 µm × 120 µm to 2.7 mm × 2.7 mm (FWHM) are available, with a flux of 1.6 × 10^16^ photons s^−1^ (measured at 325 mA ring current) in a broadband (∼5–16 keV) beam. This flux is expected to rise to 2.5 × 10^16^ photons s^−1^ at the full NSLS-II design current of 500 mA, providing an incident power density of >500 W mm^−2^ at full focus.

## Abstract 2

The development of a high-flux beamline for X-ray footprinting at NSLS-II is presented.

## 1. Introduction   

X-ray footprinting (XF) mediated by the chemical activity of hydroxyl radicals was first demonstrated as a valuable tool for examining the structure and dynamics of biological macromolecules by studies of Brenowitz, Woodson, and Chance elucidating the folding mechanism of the *Tetrahymena* ribozyme over 20 years ago (Sclavi *et al.*, 1997 ▸, 1998 ▸). Since that time, an array of XF approaches has explored a wide range of structural biology problems. XF of proteins (Kiselar *et al.*, 2002 ▸), driven by the adoption of bottom-up mass spectrometry as the ‘read-out’, has now become a leading application of footprinting to understand the structure and dynamics of proteins. The technique provides a simple, direct, and rapid way to generate hydroxyl radicals in the solution phase, where the reactivity with protein side chains serves as an accurate probe of protein solvent accessibility. Favourable characteristics of hydroxyl radicals as footprinting reagents include: small size, solvent properties similar to water, and high reactivity with amino acid side chains. In addition, no reagents beyond those required for protein stability and functional poise are necessary for the experiment since the hydroxyl radicals are generated by water radiolysis.

Exposure of aqueous biological samples to ionizing radiation leads to the near instantaneous dissociation of water into water radicals, dry electrons, and excited waters. Within 10^−12^ s, these initial products are converted into hydroxyl radicals and other primary radical species. The newly formed hydroxyl radicals diffuse through the solvent and rapidly react with the solvent-accessible side chains of amino acids, forming covalent oxidative modifications that can be identified and quantified using mass spectrometry (Takamoto & Chance, 2006 ▸; Huang *et al.*, 2015 ▸; Wang & Chance, 2017 ▸). A number of oxidative modifications are possible upon reaction of hydroxyl radicals with protein side chains. The simplest mechanism for hydroxyl-mediated protein oxidation begins with H abstraction from a side chain carbon followed by reaction with O_2_ to form a peroxyl radical, which undergoes further reactions that lead to the formation of a hydro­peroxide (+32), hydroxide (+16), or carbonyl (+14) group. Other mechanisms such as insertion of hydroxyl radicals into aromatic rings (Phe, Trp, and Tyr) or H abstraction from sulfhydryl groups (Cys or Met) are also possible. Detailed explanations of the various proposed mechanisms are beyond the scope of this paper. However, comprehensive explanations of the mechanisms and major products for each amino acid group are available in early reviews and papers on XF chemistry (Xu & Chance, 2005 ▸, 2007 ▸).

**Figure 1:**
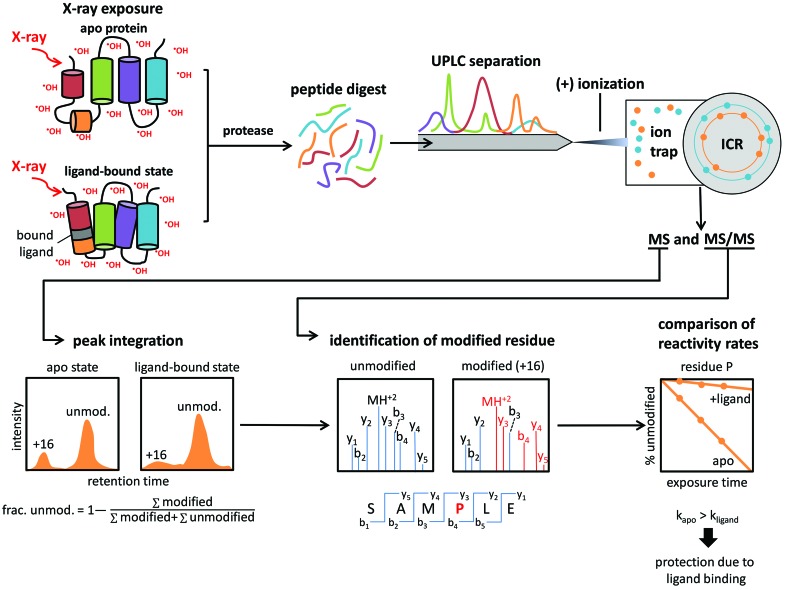
Schematic diagram of protein X-ray footprinting experiments. Control (*e.g.* apo) and experimental (*e.g.* ligand-bound) samples are exposed to an X-ray beam for microsecond to millisecond timescales. Exposed samples are digested with proteases to generate peptides, which are separated via ultra-performance liquid chromatography (UPLC). Electrospray ionization (ESI) is used to ionize the peptides, which enter the mass spectrometer where they are separated according to their mass-to-charge (*m*/*z*) ratios (MS) and are subsequently fragmented through collision with gas molecules (MS/MS). The fragmentation pattern is unique to each peptide, so MS/MS scans can be used to identify unmodified and modified peptides/residues within the sample. Extracted ion chromatograms (EICs) are used to quantify the amount of unmodified and modified peptides observed. The fraction of unmodified peptide remaining is calculated at each exposure time for each modified peptide/residue and plotted to produce dose-response curves, which are fit to a single-exponential function to determine first-order rate constants. The calculated rate constants in the control (*k*_apo_) and experimental (*k*_ligand_) samples are compared in order to determine regions of the protein that are important for function.

Protein XF experiments consist of exposing controls (*e.g.* apo protein) and different experimental samples (*e.g.* ligand-bound, complex, *etc*.) to an X-ray beam for varying exposure times. Downstream mass spectrometry (MS) analysis (Fig. 1 ▸) is conducted on each exposed sample and involves proteolytic digestion of X-ray exposed protein samples, separation of resultant peptides using ultra-performance liquid chromatography (UPLC), identification of modified and unmodified peptides using tandem mass spectrometry (MS/MS), and integration of extracted ion chromatogram (EIC) peaks to quantify the amount of unmodified and modified species for each peptide. The fraction of unmodified peptide is calculated for each exposure time and plotted to generate dose-response curves that are fit to single-exponential functions to determine first-order rate constants. Obtained rate constants can be compared across experimental conditions to monitor changes in solvent accessibility for the protein of interest in different functional and structural states.

**Figure 2:**
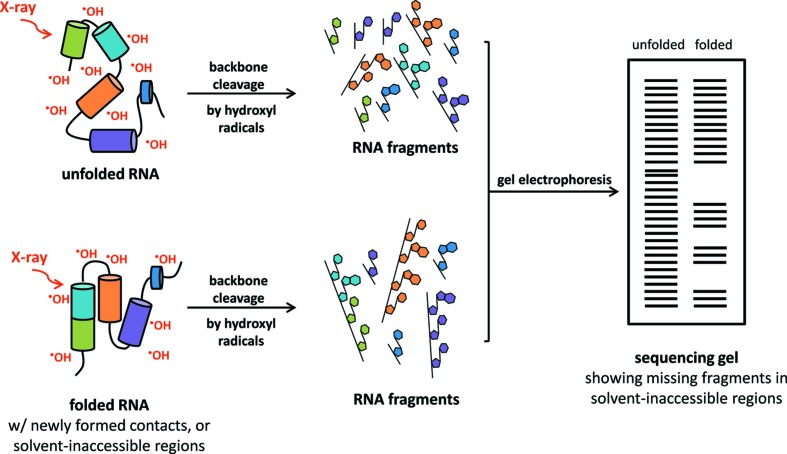
Schematic diagram of nucleic acid X-ray footprinting experiments. RNA folding experiments are an example application of X-ray footprinting. These experiments involve exposing unfolded and folded RNA samples to a focused X-ray beam, which produces hydroxyl radicals that cleave the phospho­diester backbone of the RNA molecule. The resulting nucleic acid fragments are analysed using gel electrophoresis. Missing regions observed on the sequencing gel for the folded sample indicate regions that become protected, or inaccessible to solvent (*i.e.* hydroxyl-radical cleavage), upon the unfolded RNA molecule assuming its folded state.

By contrast, in nucleic acid (NA) footprinting experiments (Fig. 2 ▸), hydroxyl radicals cleave the NA phospho­diester backbone. Consequently, the relative accessibility of the NA backbone to cleavage is dependent on the conformation of the backbone and its engagement in inter- or intra-macromolecular interactions. The NA fragments are analyzed via gel electrophoresis or sequencing, and the resulting sequencing ladder is examined for regions that are not cleaved due to protection. This straightforward, X-ray-induced, hydroxyl-radical-mediated footprinting approach has been applied to a number of structural biology studies examining ligand/drug binding, epitope mapping of monoclonal antibodies, *in vivo* studies of nucleic acid footprinting, structural waters in proteins, and membrane proteins (Hulscher *et al.*, 2016 ▸; Ralston *et al.*, 2000 ▸; Gupta, Chai *et al.*, 2014 ▸; Gupta *et al.*, 2012 ▸; Kiselar *et al.*, 2003 ▸; Adilakshmi *et al.*, 2008 ▸; Clatterbuck Soper *et al.*, 2013 ▸; Huang *et al.*, 2018 ▸; Sangodkar *et al.*, 2017 ▸).

These XF studies were performed at beamline X28C at NSLS and beamlines 5.3.1 or 3.2.1 at the Advanced Light Source (ALS). The XF beamlines at ALS are still available, but only 3.2.1, which has a considerably lower flux density than 5.3.1, is currently readily accessible to users. X28C is no longer available due to the permanent shutdown of the NSLS in 2014. Construction of the NSLS-II has been completed, and the light source is capable of delivering X-rays four orders of magnitude brighter than the NSLS. The XFP (Biological X-ray Footprinting) beamline at NSLS-II 17-BM is designed to harness the advanced capabilities of the new light source in order to produce flux densities greater than those previously available at X28C and other footprinting-capable beamlines. XFP will continue the footprinting program established by X28C, and significant effort is underway to develop new methodologies to study highly scavenging biological samples that have proven difficult to probe using previously available beamlines.

Complex biological systems such as macromolecular complexes, membrane proteins, and live cells often require high concentrations of various hydroxyl-radical-quenching reagents to preserve sample integrity, and therefore require greater radiation doses to overcome the effect of scavenging secondary reactions and to ensure sufficient radiolytic labelling of the protein sample (Gupta *et al.*, 2007 ▸). The bulk of the hydroxyl radicals generated by water radiolysis last a few microseconds due to rapid self-reaction and reaction with ‘targets’ like amino acid side chains or nucleic acid bases; additionally, the nascent hydroxyl radicals can react with buffer reagents, molecular oxygen, and other solution components leading to scavenging of hydroxyl radicals and introduction of potential secondary reactions by propagated radicals. To accumulate the needed minimal dose (such that modified products are detected), a continuous radiation dose is maintained, providing a stable steady-state concentration of hydroxyl radicals throughout the footprinting experiment. This radiation dose may range from microseconds to milliseconds, depending on the beamline and sample characteristics, and must be increased for complex biological samples due to the presence of quenching reagents such as detergents, reducing agents, or stabilizing ligands (Gupta, Celestre *et al.*, 2014 ▸; Bohon *et al.*, 2014 ▸; Sullivan *et al.*, 2008 ▸). Delivery of increased radiation dose is most easily accomplished by prolonging exposure of the sample to the X-ray beam. While increased exposure time is the simplest way to increase radiation dose, it is also the least optimal method due to damage or degradation of the protein sample caused by heat generation and secondary reactions mediated by superoxides, peroxides and other products of the radiolysis. Degradation of the sample also leads to chemical noise in LC-MS spectra, which makes it more difficult to accurately identify and quantify the observed modified and unmodified peptides. These issues can be partially circumvented by increasing the flux density of the beam, which increases the steady-state concentration of hydroxyl radicals without any need to extend exposure time (Gupta, Celestre *et al.*, 2014 ▸).

This paper describes the technical details of the design and capabilities of the XFP beamline as well as measurements of beamline performance at two distinct endstations for XF. The capillary flow (CF) endstation allows for the exposure of samples to high X-ray doses on a microsecond timescale by quickly flowing samples through a narrow capillary exposed to the highly focused X-ray beam. The multi-sample holder (MSH) endstation is used to expose 5 µl volumes of sample in PCR tubes and requires a wider, or lower flux density beam, to evenly expose stationary samples with full overlap. The CF endstation offers maximal X-ray dose delivery, while the MSH endstation offers speed and convenience since it allows for the exposure of up to 23 samples in the same amount of time (∼2–3 min) required to expose just one sample using the CF endstation. Direct beam measurements were also taken to verify the flux, size and flux-density of the beam at XFP for XF experiments in the various endstation configurations, and benchmarking experiments with a model protein were also undertaken for both endstations. Based on these results, we anticipate the improved design of XFP will enable us to perform XF experiments that will allow significant advances in the field of hydroxyl radical footprinting of macromolecules.

## 2. XFP beamline   

### 2.1. Source and optics   

**Figure 3:**
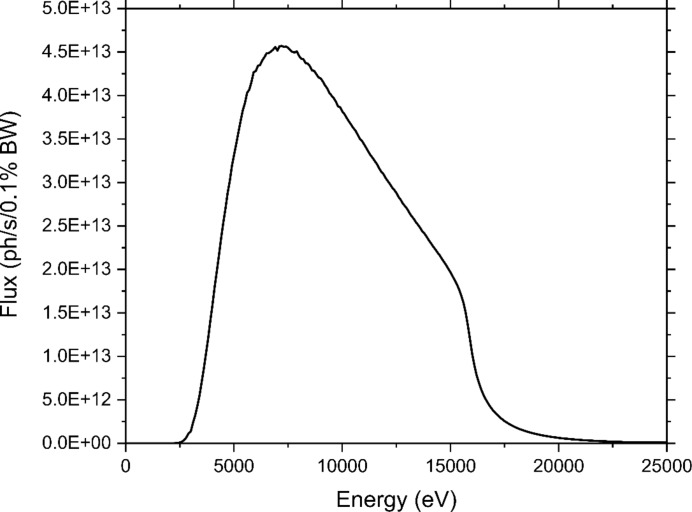
Spectral flux at XFP.

The XFP beamline is a pink beam beamline located at port 17-BM of NSLS-II on a three-pole wiggler (3PW) source that functions as a wavelength shifter (*E*_crit_ = 6.8 keV) for NSLS-II bending-magnet radiation (*E*_crit_ = 2.39 keV). The beamline accepts a 3 mrad (H) × 0.33 mrad (V) fan of radiation, focused using a 1.1 m-long rhodium (Rh)-coated toroidal mirror (Winlight X) located in the front end of the beamline at 14 m from the source, downstream from a 250 µm-thick beryllium (Be) window. The mirror substrate is single-crystal silicon (1.1 m long × 100 mm wide × 50 mm thick), with a cylindrical cut of sagittal radius 58.8 mm and a figure error of better than 1.5 µrad, designed specifically for XFP to provide 1:1 focusing at 28 m from the source. The chamber, kinematic mount and stainless steel bathtub with water cooling system were repurposed from NSLS X28C (Sullivan *et al.*, 2008 ▸). Additional changes from the X28C system include a new stand to accommodate the NSLS-II beam height, removal of the MACOR pucks, replacement of the eutectic bath with 2.98 kg of Indalloy 60 (removes tin from the system and increases the melting temperature of the eutectic), addition of absolute linear encoders (Renishaw), and replacement of the motion control system with NSLS-II Delta Tau Geobrick LV motor controllers. The toroidal mirror is set to a nominal 4.2 mrad angle and is bendable to a minimum radius of 3 km to provide meridional focusing for adjustment of the vertical beam dimension. The horizontal beam size is determined by the sample distance from the focal point within the depth of the experimental enclosure. The beamline vacuum–air interface is a water-cooled 100 µm-thick diamond window with a 10 mm open aperture. This configuration delivers >10^16^ photons s^−1^ to the sample with a broadband energy range of ∼5–16 keV and a peak flux near 7 keV. The resulting spectrum is shown in Fig. 3 ▸, calculated for the central 3 mrad (H) and 0.33 mrad (V) fan of the 3PW source using *SRW* software (Chubar *et al.*, 2017 ▸); effects of beam optics were calculated using values from the Center for X-ray Optics (CXRO, http://henke.lbl.gov/optical_constants/).

### 2.2. Photon delivery system   

**Figure 4:**
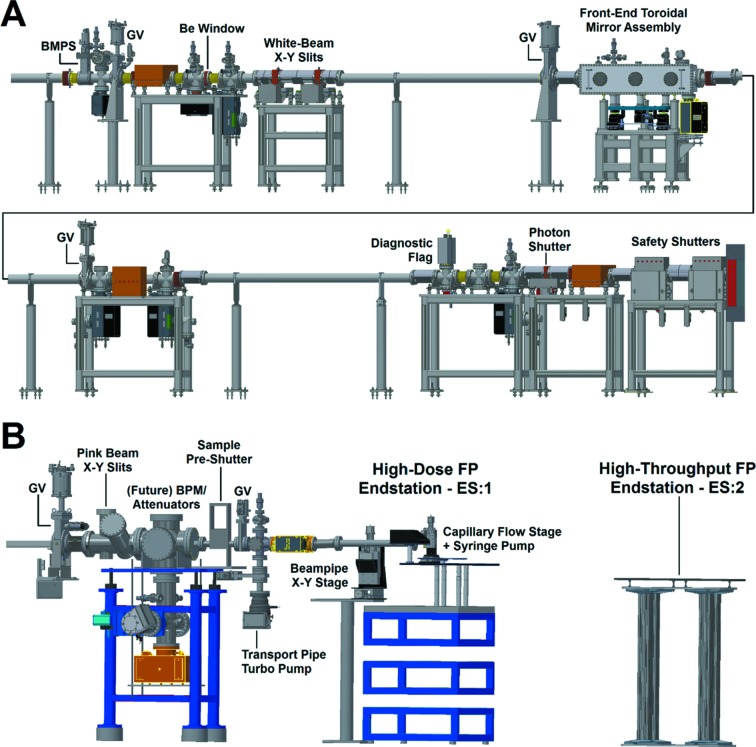
Elevation view of the XFP beamline, with the front-end components shown in panel A and the experimental hutch components shown in panel B.

In order to capture the maximum available radiation from the source, a significant portion of XFP beamline components are located inside the ratchet wall [Fig. 4(*a*) ▸]. The first component after the crotch absorber is the bending-magnet photon shutter (BMPS), followed by the first and second fixed masks and the 250 µm-thick Be window that separates the ultra-high vacuum of the storage ring from the high vacuum of the beamline. Downstream of the Be window are the white-beam slits (isolated for current measurement), the focusing mirror, two additional fixed masks, a water-cooled removable imaging screen, a photon shutter, final fixed mask, and a pair of safety shutters just upstream of the ratchet wall collimator. Along this path, two lead collimators, an inboard shadow shield, and a steel insert on top of the ratchet wall collimator allow the primary Bremsstrahlung radiation to be contained within the area inside the ratchet wall. Several gate valves are located along the beam path to allow maintenance of components; these valves are interlocked to close in the case of a vacuum leak. All beamline components downstream of the focusing mirror are shifted vertically to follow the 8.4 mrad beam angle, and the exit port of the ratchet wall at 17-BM is modified to accommodate the significantly shifted beam height. The photon delivery system downstream of the ratchet wall [Fig. 4(*b*) ▸] includes approximately 3 m of permanent components after the gate valve, consisting of beam-defining pink beam slits (Oxford Danfysik, repurposed from NSLS X6B), a chamber for anticipated in-vacuum attenuators (in the design phase at the time of writing) and beam position monitor (Sydor Instruments, in commissioning at the time of writing), a pink beam photon shutter for sample protection, ion and turbo pumps, and vacuum isolation valves. In XF configurations, the downstream beam pipe is interchangeable to provide vacuum path to two endstations at different locations within the depth of the hutch, and vacuum pumping after the valve enables rapid return to ∼10^−8^ Torr; a water-cooled diamond exit window is transferrable to the end of any of these beam pipes to provide the vacuum-to-air interface.

#### 2.2.1. Beam diagnostics   

Beam diagnostics used at XFP include both commissioning and alignment tools. The first diagnostic in the beamline is integrated into the white-beam slits upstream of the focusing mirror; the blades are isolated for current measurement, allowing observation of changes in the position of the incoming photon white beam due to accelerator adjustments. To facilitate initial beamline commissioning, a water-cooled diagnostic screen (Axilon) is installed in the front end of XFP. The screen, pneumatically actuated to move in and out of the beam path, consists of a copper wedge coated with europium-doped Y_2_O_3_ (50 µm), tilted downward at 45° with respect to the incoming beam for observation by a GigE machine vision camera (Prosilica) mounted on a viewport below. The screen saturates at beam currents higher than ∼10 mA, but was critical in initial alignment of the toroidal focusing mirror to navigate the beam through the series of fixed masks in the front-end. Subsequent alignment and beam measurement diagnostics are utilized downstream in the experimental hutch. These are not permanently installed in the beamline, with the exception of a water-cooled diamond pink beam monitor immediately downstream of the pink beam slits that is currently being commissioned. These include single-channel and quadrant diamond detectors (Bohon *et al.*, 2010 ▸), silicon diodes for use with attenuated beam, and a kilopixel diamond imaging detector for real-time beam morphology and flux measurement at the sample position during mirror adjustment to accommodate various sample requirements (Zhou *et al.*, 2015 ▸).

### 2.3. Experimental enclosure and endstations   

The experimental hutch enclosure (∼ 9.7 m × 2.5 m) (GPS, Inc.) is located immediately downstream from the ratchet wall. It contains the last 3 m of the beamline and two experimental endstations that offer alternative modes of sample delivery, or exposure, to the X-ray beam [Fig. 4(*b*) ▸]. The lead enclosure was designed according to NSLS-II standard specifications for use with white beam, with an additional lead beam stop added at the end of the enclosure to contain secondary radiation. Patch panels for BNC and Ethernet signals, as well as a user-accessible labyrinth, enable flexibility in adapting to experiment-specific user equipment. Gaseous nitro­gen, continuously provided by the facility, is also available for use. Helium is piped in from compressed gas cylinders outside of the enclosure to enable use of a helium-filled flight tube for rapid change between XF endstations when required. A mount for aluminium attenuators to control X-ray dose is provided at the end of the vacuum flight path. Two cameras on floor-stand tripods with pan–tilt–zoom capabilities are moveable throughout the hutch for observation of ongoing experiments in real time. Brief descriptions of the capillary flow (CF) and multi-sample holder (MSH) endstations at XFP are provided in Sections 2.3.1 and 2.3.2. Further details and diagrams regarding setup and operation of these types of experimental endstations can be found in previously published beamline papers (Gupta *et al.*, 2007 ▸; Gupta, Celestre *et al.*, 2014 ▸; Bohon *et al.*, 2014 ▸; Hulscher *et al.*, 2016 ▸; Hao *et al.*, 2018 ▸).

#### 2.3.1. Capillary flow (CF) endstation (ES:1)   

The toroidal mirror and capillary flow (CF) experimental endstation are placed 14 m and 28 m from the source, respectively, resulting in a 1:1 focus within the experimental hutch. Studies involving large macromolecular complexes, live cells, isolated organelles, and highly scavenging buffers require higher flux density and are conducted using the CF endstation. Samples are exposed to an X-ray beam using a 200 µm internal diameter (ID) capillary, allowing photons to be delivered to the sample over a small surface area. Apparatus for use at this endstation are mounted to a three-axis *x*-*y*-*z* motorized optical table with 1/4-inch–20 tapped holes at 1-inch spacing. The capillary exposure cell, mainly comprising a copper block with attached stainless steel slits to guide the capillary, is water-cooled to 4°C to assist in maintaining capillary integrity during exposure. The flow cell is mounted to an *x*-*y* stage with precision slides to allow careful alignment of the capillary to the beam using a diamond imaging detector placed downstream of the apparatus. The capillary is mounted vertically such that the larger horizontal dimension of the beam fully covers the width of the capillary.

For standard CF experiments, samples are loaded into a 1 mL glass gas-tight syringe, and a high-pressure syringe pump (Harvard, PHD2000) is used to push the sample through a 200 µm ID capillary exposed to the X-ray beam for varying amounts of time. Exposure time is a function of the flow rate of the sample, vertical beam size (*h*), and capillary internal ID (2*r*). The capillary ID and vertical beam size are kept constant during a given experiment, so varying the flow rate of the sample is primarily used to control the exposure time. The following equation is used to calculate the required flow rate for a given exposure time,
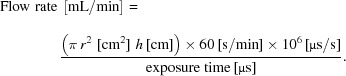
Alternatively, the vertical beam size (*e.g.* 120 × 10^−6^ m) can be divided by the desired exposure time (*e.g.* 150 × 10^−6^ s) to calculate the required speed (*e.g.* 0.8 m s^−1^) of the sample. Immediately following exposure, samples are collected in microcentrifuge tubes placed at the end of the X-ray exposed capillary. The microcentrifuge tubes are prefilled with methio­nine amide, which is added to 10 m*M* target concentration, to ensure samples are quenched immediately after passing through the X-ray beam.

This endstation is also used to host the *in vivo* exposure apparatus. For this experiment, a circulating water bath set atop a stirrer, kept at 37°C using a Haake AC200 chiller (Thermo Scientific), is used as an incubator for liquid cell culture. A positive displacement pump (Vici M50) is used to control the rate of flow of cell culture through the capillary for exposure to the X-ray beam (Hao *et al.*, 2018 ▸). A programmable fraction collector (Teledyne) is used to collect the much larger volumes of sample required (commonly 0.75 ml per tube), and enable separation of time points in time-resolved experiments. The syringe pump used for the standard capillary flow experiments can be simultaneously controlled to serve to initialize a reaction (for example, to add food to starved cells). For live cell culture exposure, the capillary exposure apparatus is generally mounted beyond the focal point to obtain a horizontal beam dimension capable of fully covering the 530 µm ID capillaries preferred for these experiments.

#### 2.3.2. Multi-sample holder (MSH) endstation (ES:2)   

XF experiments involving the exposure of small, soluble proteins in non-scavenging buffers can be exposed using far less flux density. These experiments are executed using the multi-sample holder (MSH) endstation, which allows 23 samples to be exposed in ∼2–3 min. Sample droplets (5 µL) are exposed in 200 µL PCR (polymerase chain reaction) tubes (Brandtech/FisherScientific Catalogue number 1388258), which are designed for excellent thermal transfer. This mode of sample exposure requires a wider beam to ensure that the X-ray beam spot completely covers the surface area of the sample droplet (∼2.5 mm diameter). Because the focusing mirror is set to a nominally fixed angle, the MSH experimental endstation is placed further back in the hutch (30 m from the source) in order to increase the horizontal beam width. The increased beam width reduces the flux density on the sample, and therefore millisecond exposure times (regulated by a fast shutter, see below) are necessary to deliver adequate X-ray dose to the sample. Despite the decreased flux density, MSH experiments are advantageous because they reduce the time and sample volume required for a given experiment.

For MSH experiments, PCR tubes containing sample aliquots are exposed sequentially by loading the tubes into the sample holder mounted to a precision motorized slide. An electronic Uniblitz (Vincent Associates) beam shutter controls exposure time, with a 10 ms minimum reliable exposure time. During exposure, the temperature of the sample holder is controlled according to the needs of a given experiment by a circulating water bath and Peltier coolers. The apparatus can be cooled to −30°C, which is required for maintaining nucleic acid integrity during frozen cell experiments (Adilakshmi *et al.*, 2006 ▸) and enables water–protein interactions to be studied by exposing frozen solution samples (Gupta *et al.*, 2012 ▸). After exposure, me­thio­nine amide is added to 10 m*M* target concentration in the exposed sample to quench any remaining hydroxyl radicals and prevent secondary reactions.

### 2.4. Controls   

Motor systems at XFP are driven via Delta Tau Geobrick LV motor controllers, which are the standard controller adopted by NSLS-II. Beamline controls are implemented through EPICS (Experimental Physics and Industrial Control System), Ophyd/Bluesky (Arkilic *et al.*, 2017 ▸), a Python-based data acquisition system developed by the NSLS-II Controls Group, and Control System Studio (CSS). EPICS input/output controllers (IOCs) are run on a server local to the beamline. CSS is used to access EPICS process variables (PVs), generally for beamline alignment, maintenance and troubleshooting. Ophyd translates the PVs from hardware output to a generally accessible unified program interface, which can then be used by Bluesky for a variety of experiment controls and data acquisition. Bluesky programs are used to run all endstations at XFP, with a graphical user interface for the MSH, and command-line control of the capillary flow and *in vivo* exposure systems (GUIs planned in the future). All programs are freely available on GitHub (https://github.com/NSLS-II-XFP).

### 2.5. Sample preparation facilities   

Samples for XF experiments have a variety of sample preparation requirements. Standard laboratory facilities (https://www.bnl.gov/ps/labs/) are available in attached Laboratory Office Buildings (741, 743, with 744 and 745 labs under construction); however, some samples require preparation closer to the beamline for various reasons (sample environment stability, biosafety level, short lifetime of intermediates, *etc*.). For these purposes, as well as convenience, a separate sample preparation area is located at the XFP beamline immediately downstream of the control station. This area contains several workbenches with excellent lighting, a cooling centrifuge for microcentrifuge tubes or PCR tubes, a microbalance, a UV/Vis spectrometer, a handheld fluorimeter for measurement of Alexa488 fluorescence, single and multi-channel pipettes, an incubator/shaker, and consumables required for use of this equipment. The area is relatively well separated from other beamlines at NSLS-II, which allows designation of the area (and the hutch) as acceptable for exposure of biosafety level 2 samples under controlled circumstances with appropriate preparation.

## 3. Beamline performance   

XFP was designed to provide higher radiation doses on shorter time scales compared with currently and previously available XF beamlines, with flexibility in the beam size, shape, and power density delivered to the sample. NSLS X28C, the predecessor to XFP, had the potential to reach 90 W mm^−2^ in power density (Sullivan *et al.*, 2008 ▸), but only utilized a maximum of ∼40 W mm^−2^ due to sample handling limitations; the target performance goal for XFP is 500 W mm^−2^, for an order of magnitude increase over what was previously used at X28C. To obtain high quality XF data, it is critical to ensure that the dose is deposited as uniformly as possible throughout the sample. This requirement drove the choice of energy range for the beam (∼5–16 keV), as a compromise between deposition characteristics of lower energy photons (minimal penetration) versus higher energy photons (minimal absorption) in the sample and the ability to generate radicals efficiently at sample depths of up to several millimeters. Aluminium attenuators (typically 50–1000 µm) are also used to moderate flux and change the energy spectrum of various experimental configurations. The need for a variety of available beam shapes to accommodate different sample morphologies is also apparent from the above discussion. Three such sample configurations are currently available at XFP: capillaries of 200 and 530 µm ID (for standard capillary flow and *in vivo* experiments, respectively), and a 5 µl sample held by surface tension at the bottom of a PCR tube (for MSH experiments), with a sample diameter of ∼2.5 mm. Beam measurements were made at each of these configurations, and standard samples were exposed in both capillaries and PCR tubes to verify beamline performance.

### 3.1. Beam shape, size, and power   

**Figure 5:**
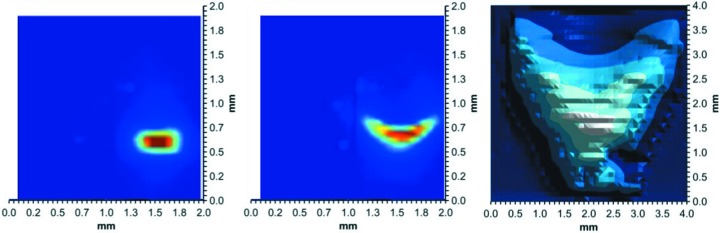
Beam profiles for use of various endstations at XFP. Left: focused beam at ∼28 m from the source (120 µm × 450 µm FWHM) for the 200 µm capillary flow apparatus. Center: focused beam (120 µm × 750 µm FWHM) for use with 530 µm capillaries for *in vivo* flow experiments. Right: unfocused beam (2.7 mm × 2.7 mm FWHM) for high-throughput experiments.

A pixelated transmission-mode diamond detector was used to monitor the beam position, shape, and flux of XFP during real-time adjustment of the toroidal focusing mirror to obtain beam shapes appropriate for capillary flow and *in vivo* XF experiments (Fig. 5 ▸, left and center, respectively). Construction and operation of the imaging detector is described elsewhere (Zhou *et al.*, 2015 ▸). To determine appropriate mirror focusing parameters for the MSH endstation, the beam required is larger than this detector, thus the beam profile (Fig. 5 ▸, right) was measured by scanning a 100 µm pinhole through the beam with a single channel detector behind the pinhole. Initial real-time adjustment of the mirror for this much larger beam was performed using a camera and fluorescent screen (copper block coated with 50 µm europium-doped Y_2_O_3_). For precision measurements of the beam size, quadrant single-crystal diamond detectors were scanned across the beam; these detectors are capable of measurement to a precision of 0.1% of the beam size. Measurements of the beam power were performed using both diamond detectors and via copper block calorimetry. The highest power density beam directly measured at the time of writing was 290 W mm^−2^, with 15.7 W in a beam size of 450 µm (H) × 120 µm (V) (FWHM). The measurement was made at a ring current of 325 mA, 210 mm from the exit window and inside of a nitro­gen-filled enclosure to protect the detector from ozone corrosion. Standard CF sample positioning is less than 100 mm from the exit window; the estimated corresponding power density delivered to the actual sample position is thus 345 W mm^−2^ (calculated using the XFP spectrum and optical constants obtained from CXRO), with a total flux of 1.6 × 10^16^ photons s^−1^ based on the spectrum (Fig. 3 ▸). When NSLS-II achieves the design goal of 500 mA beam current, XFP is thus expected to be capable of delivering up to >500 W mm^−2^ to the focal position at 28 m from the source, with a total flux of ∼2.5 × 10^16^ photons s^−1^. This power density is >5-fold greater than was previously possible (and >10-fold greater than was employed) at X28C (Sullivan *et al.*, 2008 ▸; Bohon *et al.*, 2014 ▸), and meets the design goals of the beamline.

### 3.2. Alexa 488 fluoro­phore measurement   

To further evaluate beamline capability, the degradation rate of Alexa 488 fluoro­phore was used as a pr­oxy for radiation dose in the sample. With increased exposure to an X-ray beam, Alexa fluorescence decreases with first-order kinetics, so calculated rates for Alexa 488 degradation can serve as a relative measurement of radiolytic dose (Gupta *et al.*, 2007 ▸). All Alexa dose-response experiments are performed at room temperature due to the temperature sensitivity of the dye, and exposed samples are immediately analysed following exposure using a Turner Biosystems TBS-380 fluoro­meter. The fraction of remaining fluorescence is calculated and plotted versus exposure time, and the resulting rate constant is used to compare the efficiency of hydroxyl radical formation under the different experimental conditions.

**Figure 6:**
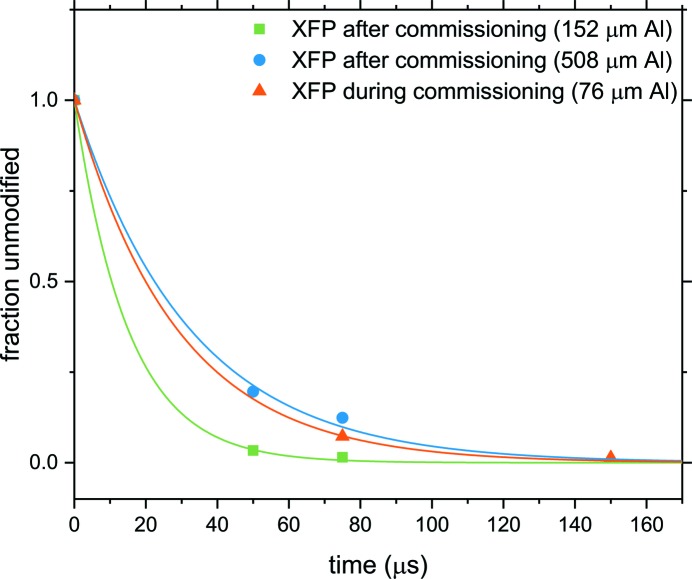
Dose-response curves for NSLS-II XFP at optimal beam focus based on Alexa 488 fluorescence decay in 10 m*M* sodium phosphate buffer (pH 7.4) upon X-ray exposure using a high-speed capillary flow (CF) system. During commissioning, a rate of 35000 s^−1^ was obtained with 76 µm Al attenuation and a beam current of 250 mA. Alexa studies completed after commissioning at 400 mA ring current resulted in rates of 67000 s^−1^ and 31000 s^−1^ using 152 µm Al and 508 µm Al, respectively.

The maximum rate (67000 s^−1^) measured for Alexa 488 fluoro­phore exposed using high-speed capillary flow at XFP was 229-fold greater than the rate published for SOLEIL, 33-fold higher than the highest rate published for NSLS X28C, and 4.9-fold greater than the maximum rate published for beamline 5.3.1 at ALS (Table S1 of the supporting information) (Gupta *et al.*, 2007 ▸, 2014 ▸; Baud *et al.*, 2017 ▸). It should be noted that this value was attained using a 152 µm aluminium (Al) attenuator, while the analogous measurements at other beamlines were conducted in air or vacuum. Thus, the comparable un-attenuated rates for XFP using this assay would be considerably higher. Fig. 6 ▸ shows the dose-response curves for Alexa 488 decay fluorescence both during and after commissioning of XFP. Measuring maximal Alexa fluorescence decay rates is challenging on high-flux beamlines (*e.g.* NSLS-II XFP, NSLS X28C, ALS 5.3.1, *etc*.) because they produce high steady-state concentrations of hydroxyl radicals on short timescales, leading to near total quenching of Alexa 488 fluorescence within the first few exposure time points. As seen here, attenuation can be used to bring the dose into range. Given the 500 mA design beam current for NSLS-II, alternative improved hydroxyl radical dosimetry assays will need to be pursued for high-dose XF experiments.

### 3.3. X-ray footprinting of cytochrome *c* as a standard sample   

Cytochrome *c* is an excellent benchmark sample because it is a small, soluble protein with a well characterized structure and function, which has successfully been used to assess the capabilities of beamlines at the APS (10-BM-A), ALS (8.3.2, 5.0.2, and 5.3.1), CHESS (A2), and, most recently, NSLS (X28C), NSLS-II (17-BM) (Bohon *et al.*, 2014 ▸). Similar proteins (*e.g.* ubiquitin, myoglobin, *etc*.) can serve as equally effective benchmark samples, but cytochrome *c* has been chosen for beamline scientific commissioning because it allows accumulation of historical data that simplifies mass spectrometry data analysis and comparison of beamline performance.

#### 3.3.1. Cytochrome *c* irradiation   

Rabbit cytochrome *c* (UniprotKB P00008) was purchased from Sigma-Aldrich and diluted in phosphate-buffered saline (PBS; 1 m*M* KH_2_PO_4_, 3 m*M* Na_2_PO_4_, 155 m*M* NaCl, pH 7.4) (ThermoScientific) to a final concentration of 5 µ*M*. For MSH experiments, 5 µL aliquots were exposed for 10–30 ms at room temperature and were pooled to collect a total of 25 µL for each exposure time. The X-ray beam used for MSH experiments was >2.5 mm in diameter and was attenuated with 762 µm of Al, with an NSLS-II storage ring current of 300 mA. For CF experiments, 100 µL of 5 µ*M* cytochrome *c* was exposed for 75–300 µs. The size of the beam used for capillary flow experiments was 450 µm × 120 µm, with storage ring current of 250 mA. The total X-ray dose of the beam was attenuated using 508 µm Al. Immediately following exposure, samples were quenched with a final concentration of 10 m*M* me­thio­nine amide, flash frozen using liquid nitro­gen, and stored at −80°C until mass spectrometry analysis.

#### 3.3.2. Mass spectrometry analysis   

For all samples, 1 µg of cytochrome *c* was reduced and alkyl­ated by incubation with 10 m*M* DTT for 1 h at 37°C followed by incubation with 25 m*M* iodo­acetamide for 30 min at room temperature under dark conditions. After reduction and alkyl­ation, the samples were digested with Lys-C for 3 h at 37°C followed by overnight trypsin digestion at 37°C. Both proteases were used in a 1:10 (*w*/*w*) enzyme:protein ratio. Peptides were analyzed by a ThermoScientific Orbitrap Elite hybrid ion trap mass spectrometer interfaced with a Waters nanoACQUITY UPLC according to standard procedures.

#### 3.3.3. Data analysis   

MS/MS spectra were manually validated using *MassMatrix* software (Xu & Freitas, 2009 ▸). The abundance of unmodified and oxidized species for each peptide was determined by extracting and integrating their selected ion chromatograms using *XCalibur* software from Thermo Fisher Scientific. The fraction of unmodified peptide for each exposure time was calculated based on previous approaches (Takamoto & Chance, 2006 ▸). Dose-response curves were generated by plotting the fraction of unmodified peptides versus attenuation level, and the curves were fit to a single exponential equation using *Origin 8*. The equation is described by
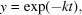
where *k* is the rate of modification in s^−1^ and *t* is the exposure time in s.

#### 3.3.4. Results   

**Figure 7:**
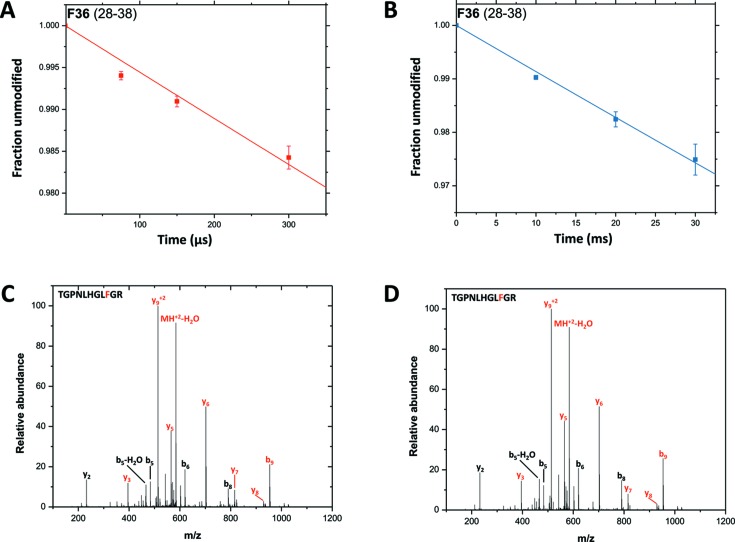
Example dose-response curves (A, B) and MS/MS scans (C, D) are shown for F36 in both the CF (A, C) and MSH (B, D) datasets.

MSH and CF data generated using XFP were compared with results from previous beamline experiments (Bohon *et al.*, 2014 ▸). In previous benchmarking experiments, 11–13 modified residues were observed for cytochrome *c*, while, for experiments conducted on XFP, 11 and 14 modified residues (Fig. S1) were identified for cytochrome *c* exposed using capillary flow and MSH, respectively. CF and MSH benchmarking experiments both resulted in reproducible, radiolytic labelling of the sample, and all dose-response plots for both datasets are available in the supporting information (Figs. S2 and S3). Dose-response plots of the residue F36 are shown as an example [Figs. 7(A) and 7(B) ▸]. The identity of F36 was confirmed via manual validation of MS/MS scans from each of the extracted ion chromatograms (EICs) belonging to the residue [Figs. 7(C) and 7(D) ▸].

Signal-to-noise (S/N) ratios of the EICs were also examined in order to assess data quality. The S/N ratios for the unmodified and modified extracted ion peaks for peptide 28–38 were determined for the 75 µs and 30 ms exposures of the CF and MSH data, respectively. These exposure times were chosen for S/N analysis because they had roughly equivalent X-ray doses based on Alexa dose-response studies showing ∼38% fluorescence remaining following X-ray exposure (Fig. S4). The calculated S/N ratios (Fig. S5) for both sets of data are quite large (*i.e.* >20), which ensures that the peak areas of the extracted ion peaks can be accurately and reproducibly quantified. This demonstrates that MSH and CF are both effective methods for exposing small, soluble proteins in non-scavenging buffers.

However, the exposure of complex, scavenging samples using the MSH endstation may require exposure times much greater than 30 ms. The longer exposure times could potentially lead to a marked deterioration in MS data quality, making it difficult to quantify the unmodified and modified species of a particular peptide (Bohon *et al.*, 2014 ▸; Gupta, Celestre *et al.*, 2014 ▸; Gupta *et al.*, 2007 ▸). Fig. S6 shows SDS-PAGE gels of cytochrome *c* samples exposed via MSH using progressively lower beam attenuations, or increased flux, while keeping exposure time constant. Sample degradation is apparent after the first time point in the samples receiving the higher X-ray doses as shown by the diffuse, barely visible bands on the gel. CF provides a way to deliver high X-ray doses to samples without sacrificing data quality because a small, high flux density beam can be used to deliver a high X-ray dose to samples on a microsecond timescale, limiting sample degradation caused by secondary reactions.

**Figure 8:**
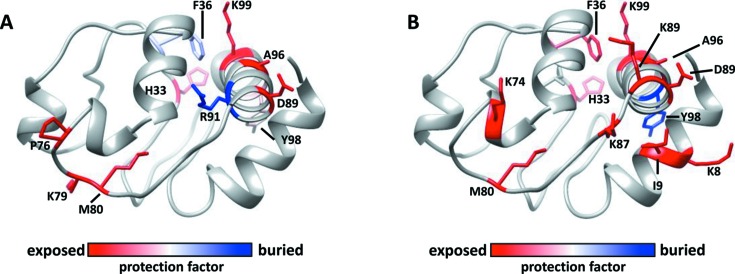
Calculated protection factor values mapped onto the structure of rabbit cytochrome *c* are shown for F36 in both CF (A) and MSH (B) datasets.

To further compare the MSH and CF data obtained on XFP, protection factors (PFs) for each modified residue were calculated by dividing each residue’s rate of modification (listed in Tables S2 and S3) by its intrinsic reactivity to hydroxyl radicals (Huang *et al.*, 2015 ▸). The rate of modification is a function of both a side chain’s solvent accessibility and intrinsic reactivity with hydroxyl radicals, so the calculation of PF values allows for the comparison of side chain solvent accessibility within a given protein on an absolute scale (Huang *et al.*, 2015 ▸; Xu & Chance, 2005 ▸). The PF values for the residues in each dataset were mapped onto the structure of rabbit cytochrome *c* (Fig. 8 ▸) in order to create a visual representation of the regions within the protein that appear to be buried (*i.e.* less solvent accessible) or exposed (*i.e.* more solvent accessible). Both the MSH and CF data show a similar pattern of solvent accessibility with the majority of the observed modifications being highly solvent accessible, or located on the surface of cytochrome *c*.

**Figure 9:**
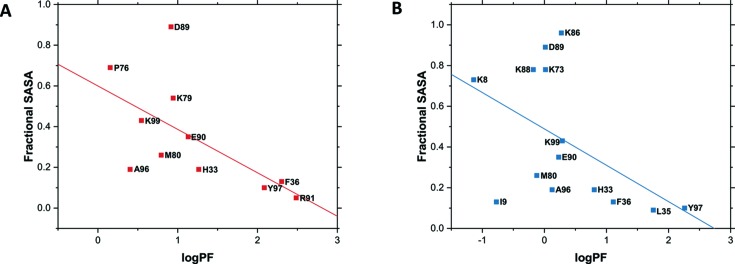
Representative scatter plots of logPF plotted against fractional SASA for the multi-sample holder (MSH) and capillary flow (CF) experimental data. The correlation coefficients for the CF (A) and MSH (B) data were −0.58 (*p*-value < 0.05) and −0.55 (*p*-value < 0.05), respectively.

Mapping the PFs onto the structure of rabbit cytochrome *c* only provides a qualitative assessment of how well the calculated protection factors correspond to the computed solvent-accessible surface area (SASA) of the amino acid side chains within the structure of cytochrome *c*, but a more quantitative measurement can be performed by determining the correlation coefficient for the scatter plot between logPF and the fractional SASA of the modified residues. The scatter plot for the MSH data is shown as an example and was found to have a correlation coefficient (ρ) of −0.55 to −0.58, *p*-value < 0.05 (Fig. 9 ▸), well within expectation based on previous studies (Huang *et al.*, 2015 ▸). Thus, the ρ obtained for our data establishes that our beamline setup and exposure parameters can provide accurate absolute information about the solvent accessibility of residue side chains within an intact, native protein.

## 4. Summary and outlook   

NSLS-II XFP at 17-BM provides significantly higher flux density for XF studies than has previously been available, on track to exceed 500 W mm^−2^ (4.6 × 10^17^ photons s^−1^ mm^−2^) as NSLS-II reaches its full design current. Beam sizes from hundreds of micrometers to several millimeters, provided at two alternate endstation locations, allow XFP to adapt to multiple XF sample morphologies. Benchmarking experiments using a standard protein (cytochrome *c*) establish that accurate and reproducible in-solution XF structural data can be obtained using either the CF or MSH experimental endstations, providing users with flexibility in designing and conducting XF experiments. In the first year of the general user program at XFP, the beamline has already hosted XF experiments on viruses, live cells, organelles, protein complexes resistant to crystallization, pharmaceuticals binding with targets, peptide–nanoparticle interactions, prions (Li *et al.*, 2018 ▸), time-resolved studies (Du *et al.*, 2019 ▸) and RNA-protein complexes, as well as more standard protein–protein interaction studies. Many of these studies were not feasible without the capabilities of XFP, and first science results are eagerly anticipated. We hope to encourage further innovation by optimizing sample delivery/exposure and adding complementary structural techniques to provide further insight into structure and function of biological macromolecules. Additional capabilities currently being developed at XFP include time-resolved footprinting apparatus and an ultra-high-throughput endstation that will allow users to expose 96 samples in as little as 4 min. An additional endstation downstream of the XF endstations, currently intended for experiments requiring monochromatic radiation such as X-ray absorption spectroscopy, will be detailed in future work. NSLS-II XFP is the premier beamline for X-ray footprinting in the world, and we expect the expanded capabilities and continued upgrades of XFP endstations to enable significant contributions in the field of structural biology not possible with XF five to ten years ago. Potential users interested in performing XF experiments are encouraged to contact the corresponding authors to discuss the feasibility of their proposed experiments and to visit our webpage (https://www.bnl.gov/nsls2/beamline/17-BM) to learn more about the requirements and process for submitting a general user proposal to NSLS-II.

## Supplementary Material

Tables S1 to S3; Figs. S1 to S6. DOI: 10.1107/S1600577519003576/ig5064sup1.pdf
