# Supplementary material for: The XFP (17-BM) beamline for X-ray footprinting at NSLS-II
Source: J Synchrotron Radiat. 2019 Jun 4;26(Pt 4):1388–99. doi: 10.1107/S1600577519003576 (PMC6613119; doi:10.1107/S1600577519003576)
Supplement: Supplementary file 1 [file s-26-01388-sup1.pdf]

JOURNAL OF
SYNCHROTRON
RADIATION

Volume 26 (2019)

Supporting information for article:

The XFP (17-BM) Beamline for X-ray Footprinting at NSLS-II

Awuri Asuru, Erik R. Farquhar, Michael Sullivan, Donald Abel, John Toomey, Mark R. Chance and Jen Bohon

Table S1 Published rates reported Alexa rates for beamlines capable of performing X-ray footprinting experiments (Bohon *et al.*, 2014; Gupta, Celestre *et al.*, 2014; Baud *et al.*, 2017). Note that rates for XFP were obtained using either 76 µm Al (during commissioning) or 152 µm Al (after commissioning) attenuation, which was necessary in order to obtain data above the limit of detection of the fluorometer.

Facility	Beamline	Max Alexa rate (s^{-1})	Data Originally Published
NSLS-II	XFP (after commissioning)	67000	this work
NSLS-II	XFP (during commissioning)	35000	this work
NSLS	X28C	2034	Bohon *et al.* 2014
ALS	5.3.1	13764	Gupta, Celestre *et al.*, 2014
ALS	3.2.1	55	Bohon *et al.*, 2014
ALS	8.3.2	35.8	Bohon *et al.*, 2014
ALS	5.0.2	1.17	Bohon *et al.*, 2014
APS	10-BM-A	82.1	Bohon *et al.*, 2014
SOLEIL	Metrology	292.1	Baud *et al.*, 2017

A 001 GDVEKGKKIF VQKCAQCHTV EKGGKHKTGP NL**H**GL**F**GRKT GQAVGFSYTD 050

051 ANKNKGITWG EDTLMEYLEN PKKYI**P**GT**KM** IFAGIKKK**DE** **R**ADLI**AY**L**K**K 100

101 ATNE 104

B 001 GDVEKGK**KI**F VQKCAQCHTV EKGGKHKTGP NL**H**G**LF**GRKT GQAVGFSYTD 050

051 ANKNKGITWG EDTLMEYLEN PK**K**YIPGTK**M** IFAGI**K**K**KDE** RADLI**AY**L**K**K 100

101 ATNE 104

Figure S1 Peptide map for CF (A) and MSH (B) data showing observed peptides (black underlined) and modified residues (bold red).

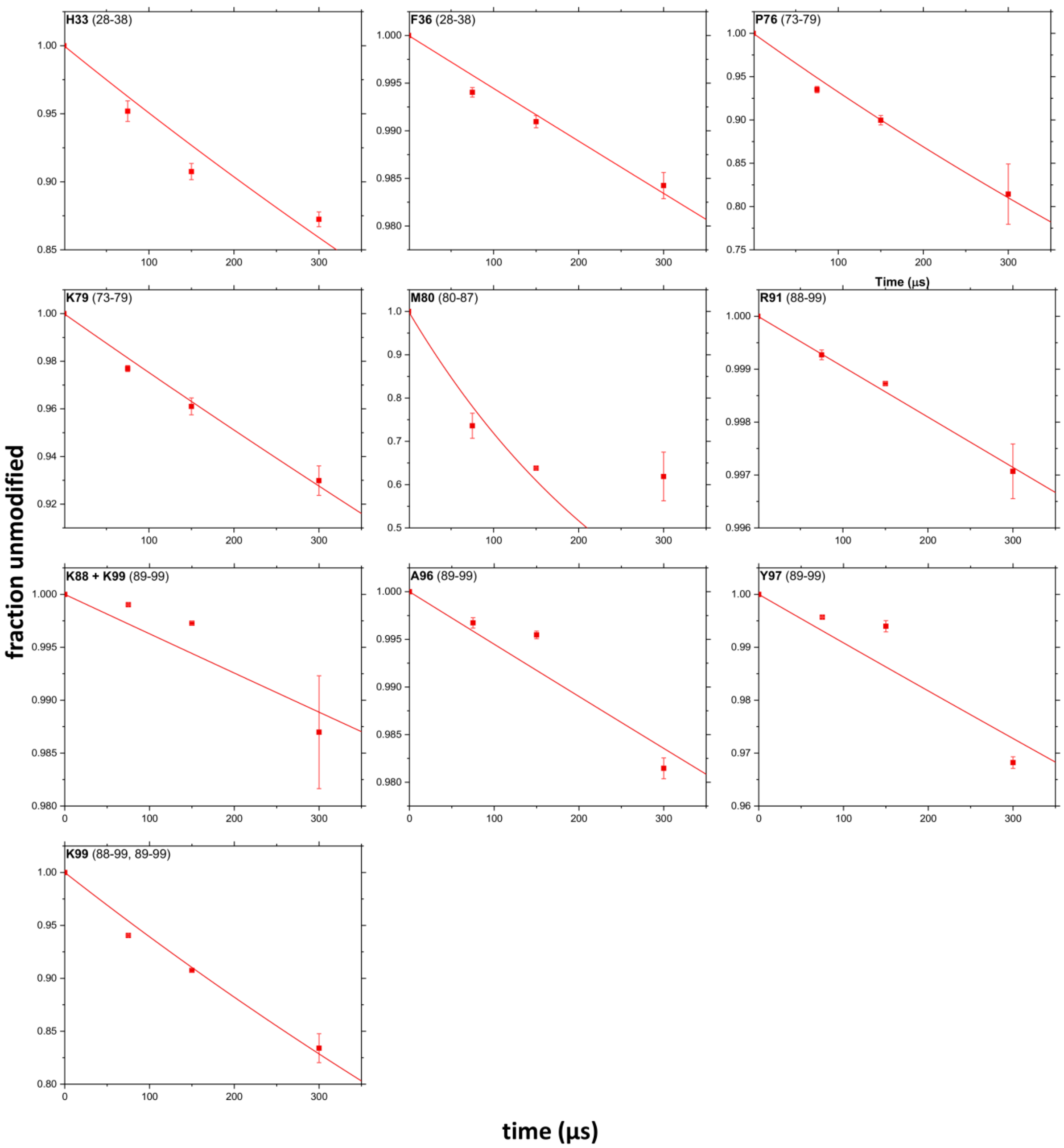

Figure S2 Dose-response plots for capillary flow (CF) cytochrome c benchmarking experiments.

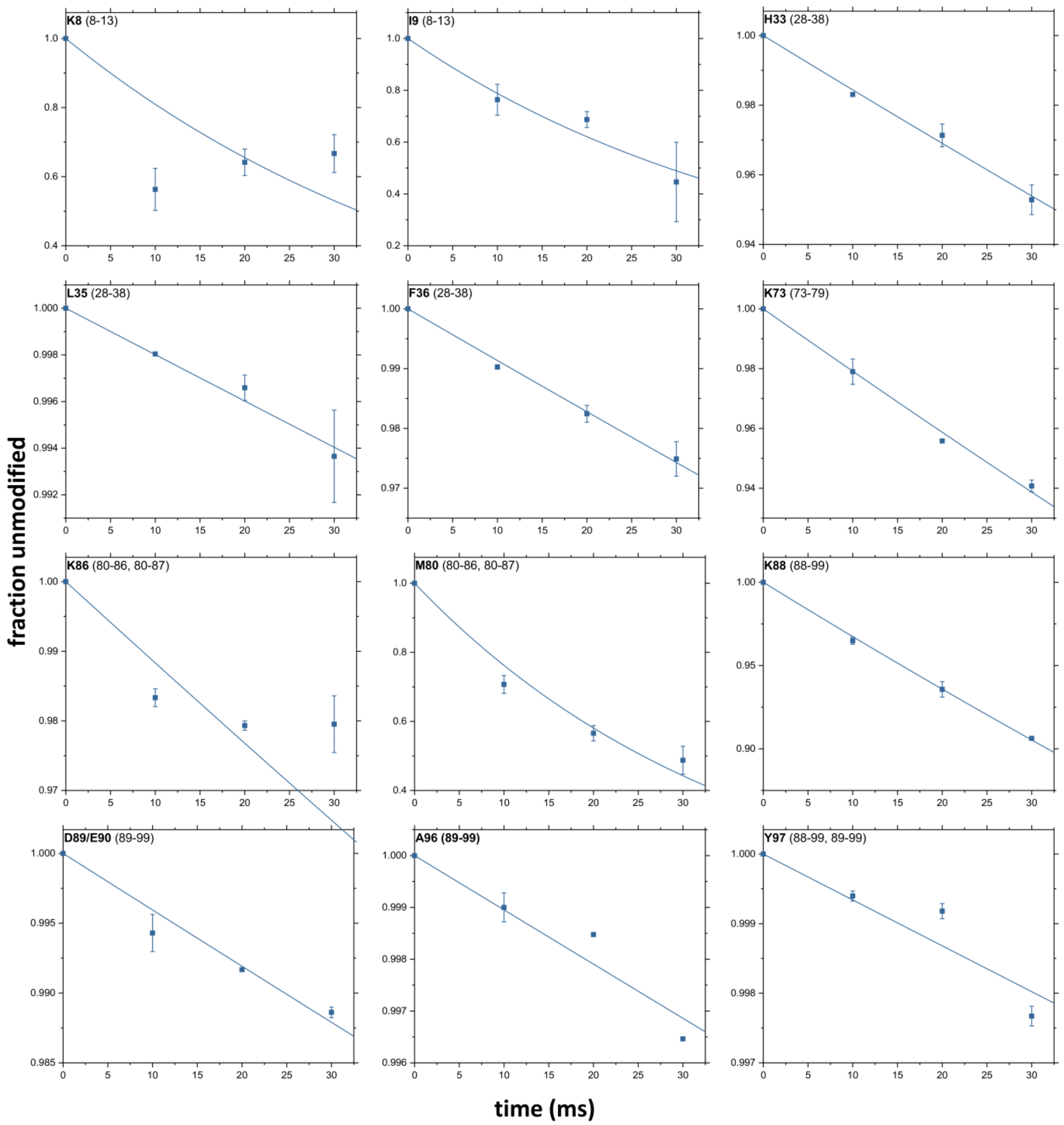

Figure S3 Dose-response plots for multi-sample holder (MSH) cytochrome c benchmarking experiments.

Table S2 Calculated rates of modification and protection factors for observed modified residues in CF benchmarking experiments.

Peptide	Modified residue	Rate (ms^{-1}) x10^{-3}	Intrinsic reactivity	PF
28-38	H33	510	10.00	19.61
28-38	F36	56	11.20	200.00
73-79	P76	700	1.00	1.43
74-79	K79	250	2.20	8.80
80-87	M80	2200	20.50	9.32
88-99	R91	9.5	2.90	305.36
88-99	K88+K99	37	4.40	118.92
88-99<br>89-99	K99	630	2.20	3.51
89-99	A96	55	0.14	2.55
89-99	Y97	92	12.00	130.43
89-99	D89/E90	51	0.42	8.24 (D89)<br>13.53 (E90)

Table S3 Calculated rates of modification and protection factors for observed modified residues in MSH benchmarking experiments.

Peptide	Modified residue	Rate (s^{-1})	Intrinsic reactivity	PF
8-13	K8	30.34	2.20	0.07
8-13	I9	26.08	4.40	0.17
28-38	L35	0.16	9.30	56.95
28-38	F36	0.87	11.20	12.91
28-38	H33	1.57	10.00	6.37
73-79	K73	2.11	2.20	1.04
88-99	K88	3.32	2.20	0.66
89-99	D89/E90	0.41	0.42	1.04 (D89)<br>1.70 (E90)
89-99	A96	0.10	0.14	1.34
89-99	K99	1.13	2.20	1.95
80-86, 80-87	K86	1.17	2.20	1.88
80-86, 80-87	M80	27.16	20.50	0.75
88-99, 89-99	Y98	0.07	12.00	181.88

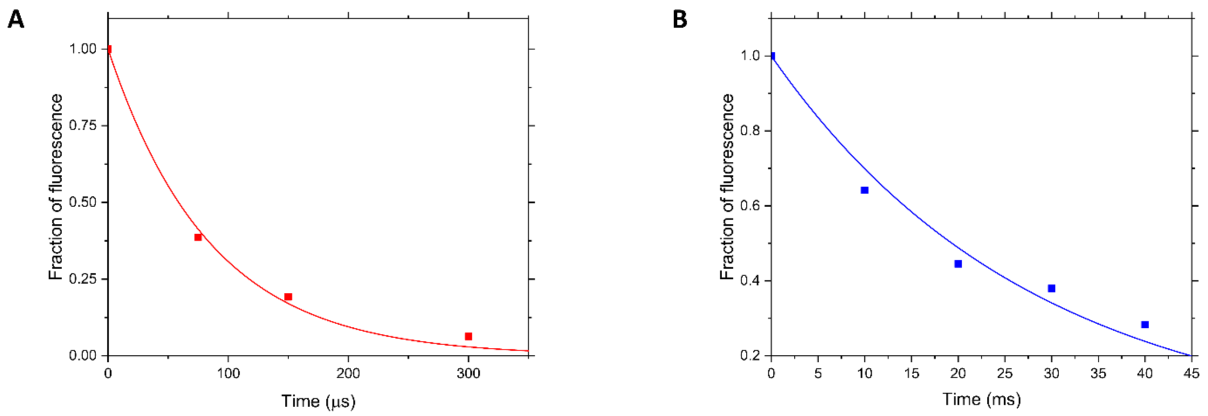

Figure S4 Dose-response plots of Alexa 488 decay in the presence of 5 μM rabbit cytochrome c upon X-ray exposure using the CF (panel A, k = 11795 s^{-1}) and MSH (panel B, k = 32 s^{-1}) experimental endstations.

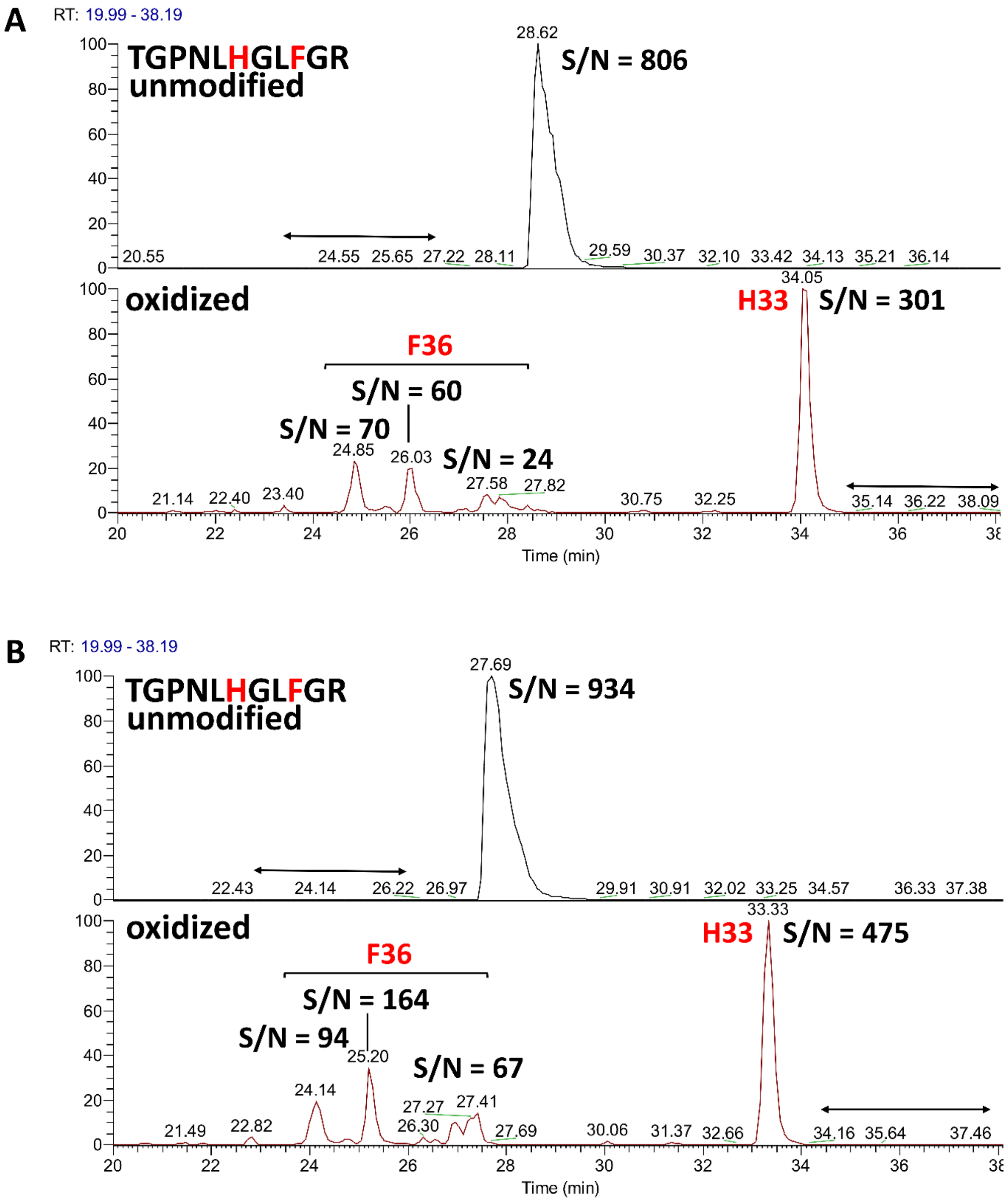

Figure S5 Extracted ion chromatograms (EICs) from the CF (A) and MSH (B) datasets were used to calculate the signal-to-noise ratios for the unmodified and +16 modified (F36 and H33) species for peptide 28-38. The EICs are labelled with the signal-to-noise ratios, and the arrows indicate the regions used to estimate the spectral noise.

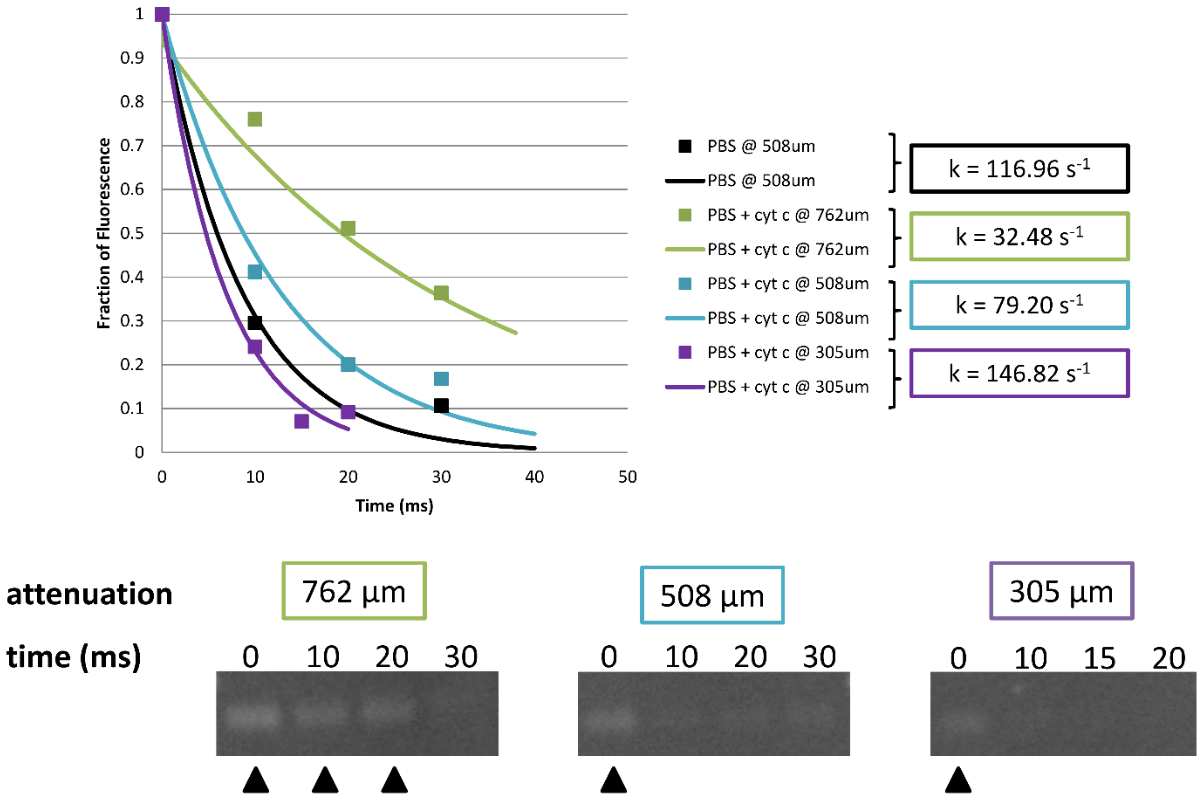

Figure S6 Sample degradation as a function of X-ray dose measured using SDS-PAGE gels. The black arrows indicate bands of interest.
